# Use of Infrared Thermography during Ejaculation Process and Its Link with Semen Quality and Freezability in Dogs

**DOI:** 10.3390/ani11113023

**Published:** 2021-10-20

**Authors:** Koray Tekin, Muhammed Enes İnanç, Doğukan Özen, Beste Cil, Kemal Tuna Olğaç, Burak Yılmaz, Umut Taşdemir, Pürhan Barbaros Tuncer, Serhat Büyükleblebici, Ali Daşkın, Ongun Uysal, Calogero Stelletta

**Affiliations:** 1 Department of Reproduction and Artificial Insemination, Faculty of Veterinary Medicine, Ankara University, Ankara 68100, Turkey; tekin.koray@hotmail.com (K.T.); cil.beste@gmail.com (B.C.); ktolgac@gmail.com (K.T.O.); vethek.burakyilmaz@gmail.com (B.Y.); ali.daskin@ankara.edu.tr (A.D.); ongun.uysal@veterinary.ankara.edu.tr (O.U.); 2 Department of Reproduction and Artificial Insemination, Faculty of Veterinary Medicine, Burdur Mehmet Akif Ersoy University, Burdur 15030, Turkey; enesinanc@hotmail.com; 3 Department of Biostatistics, Faculty of Veterinary Medicine, Ankara University, Ankara 06560, Turkey; dogukanozen@yahoo.com; 4 Department of Reproduction and Artificial Insemination, Faculty of Veterinary Medicine, Aksaray University, Aksaray 68100, Turkey; tasdemiru@gmail.com; 5 Technical Sciences Vocational School, Mersin University, Mersin 33110, Turkey; barbarostuncerp@mersin.edu.tr (P.B.T.); serhatb@mersin.edu.tr (S.B.); 6 Department of Animal Medicine, Production and Health, University of Padova, 35122 Padova, Italy

**Keywords:** CASA, ejaculation, infrared thermography, Malakli dog, semen freezability

## Abstract

**Simple Summary:**

Scientific attention to infrared technology has grown over the last decade. Remote and non-invasive monitoring techniques are of great importance in discovering ejaculation response and future trends because of their role in vascular flux regulation. However, detailed information about its use in andrology has yet to be fully explained. Therefore, we aimed to reveal information about the amount of sperm to be obtained by observing stress levels with non-invasive eye temperature measurement, and the relationship between various reproductive temperature patterns and parameters of the animal’s various physiological conditions such as age, body condition, total ejaculation time and testicular volume.

**Abstract:**

This study aimed to describe the thermal variation of external reproductive tracts during ejaculation in relation to sperm quality in dogs. Forty-six adult fertile dogs were monitored using a thermal camera before, during and after the semen collection, taking into account penile and scrotal temperatures as reproductive thermal patterns while eye and perianal temperatures were recorded as complementary thermal patterns of behavioral response. The parameters were classified depending on age (≤4 years and >4 years), body weight (BW) (≤75 kg and >75 kg), sperm concentration (CON) (≤300 million and >300 million), total testicular volume (TTV) (≤600 cm^3^ and >600 cm^3^) and total ejaculation time (TET) (≤800 s and >800 s) of the animals from which semen was collected successfully. Heavier males (*p* < 0.05) that have more consistent testicles (*p* < 0.01) as well as quicker ejaculate responders (*p* < 0.001) and lower scrotal temperature had better semen (Δ motility) freezability. The lower eye temperature prior to the ejaculation (*p* < 0.01), lower scrotal temperature following ejaculation (*p* < 0.01), and conversely, higher penile temperature during the ejaculation (*p* < 0.001) had a higher sperm concentration. Furthermore, the sperm freezability was negatively correlated with total ejaculation time (r = −0.39, *p* < 0.05) and sperm abnormalities were lower in the ejaculate of dogs having a higher temperature of the scrotum, bulbus and penis. In conclusion, infrared monitoring throughout semen collection in dogs can provide information on behavioral reactions during human manipulation, as well as semen quality and testicular functionality.

## 1. Introduction

The Malakli is a giant aggressive guard dog breed (90 kg average body weight) that derives the name from the dropped lips. This dog breed is especially used to protect small ruminant herds from predators in open pastures. To protect this breed, semen freezing study is carried out in order to preserve gamete cells by this group for the first time. 

Infrared thermography (IRT) presents a remote and non-invasive technique that measures the surface temperature by either emitting or detecting radiant heat in the infrared part of the electromagnetic spectrum of every object. Thereby, with the aid of IRT, it is possible to detect even minimal temperature variations [1,2,3]. Infrared thermography is common practice for different purposes in internal medicine and surgery, particularly as a diagnostic tool in small animals [4,5,6,7,8]. However, in reproduction, there are few reports related to pregnancy diagnosis, estrus detection, mastitis, or to the development of mammary tumors [8,9,10,11,12,13]. Infrared thermography in clinical animal andrology is considered in various species and specific information about its use for the definition of a thermal pattern of scrotal surface temperature is present above all in species with a pendulous scrotum [14,15,16,17,18,19,20,21].

For testicles to function optimally, the temperature should not exceed 33–34.5 °C since the increased temperature has a negative effect on spermatogenesis [17,18,19,20]. The regulation of this temperature depends on the contraction of muscles, sweat glands activity, heat irradiation of the surface and arteriovenous heat exchange by the pampiniform plexus counter-current mechanism [22]. Interestingly, the scrotal temperature has a completely inverse variation during erection and ejaculation. Erection is under parasympathetic control (muscarinic receptors), which causes cavernous tissue to fill with blood and the penis to become erect [23]. Ejaculation is under sympathetic control (alfa adrenergic receptors), which causes the contractions of ischiocavernosus and bulbocavernosus muscles [24]. When the inner thigh or reproductive organ is stroked during the copulation or semen collection, a superficial reflex that is called cremasteric reflex occurs. This condition takes place during a fight, flight and sexual arousal as well [17,25]. During erection, penile surface veins become contracted and thus the surface temperature of the penis decreases. However, with erection, an increase of the scrotal surface temperature occurs due to the cremasteric reflex, particularly at the area of the epididymis and it decreases below its basal temperature during the ejaculation, wherein less than a minute following the ejaculation, it increases back to its basal temperature [26]. Similarly, a short-lasting increase in heart rate as well as a slight increase in body temperature, known as stress-induced hyperthermia, reflect a defensive response to physiological and psychological stress factors such as handling, introduction into a new environment [27], and conditioned fear in various species [28,29,30]. As a result, the muscles and the central nervous system warm up, which could be beneficial during the “fight or flight” reaction to potentially threatening stimuli [31]. Similarly, alterations in the blood flow affect the temperature of the eyelids and the lacrimal area. Particularly, in the event of acute stress, the temperature of the lacrimal caruncle increases rapidly as a sympathetic response of the autonomic nervous system to prepare the individual for a fight or flight situation [32,33,34]; while in the presence of psychogenic stressors, passive responses such as freezing occur under the control of the parasympathetic system with the delayed activation of the hypothalamic–pituitary–adrenal axis [35,36]. Owing to this underlying physiology, IRT has become an acceptable tool for detecting psychological stress in dogs [37]. However, specific limitation factors have to be considered such as the distance between the ROI and the camera [38], the coat color, quality and thickness [39], as well as contamination, wounds and moisture [9,39] significantly affect the outer surface temperature of the body. Physical activity increases the body temperature through muscle contraction, and thus the whole-body surface temperature [40]. These limitations can only be avoided with thermal gathering through defined ROIs and the analysis of the variations in heat production.

Therefore, we aimed to integrate the use of IRT throughout semen collection to define the thermal patterns of the ejaculation concerning the semen quality and freezability, as well as various physiological and reproductive traits of the dogs that could affect the thermal response, as age, body weight (BW), total testicular volume (TTV), total ejaculation time (TET) and sperm concentration (CON). In light of this knowledge, it is hypothesized that thermal patterns of the external reproductive organs can differ between the flaccid and erectile states during, before and after ejaculation.

## 2. Materials and Methods

### 2.1. Ethics

Animal experiments were conducted according to ethical principles and were approved by the Animal Ethics Committee of Ankara University (2015-21-230).

### 2.2. Location, Environmental Conditions, and Animals

**Figure 1:**
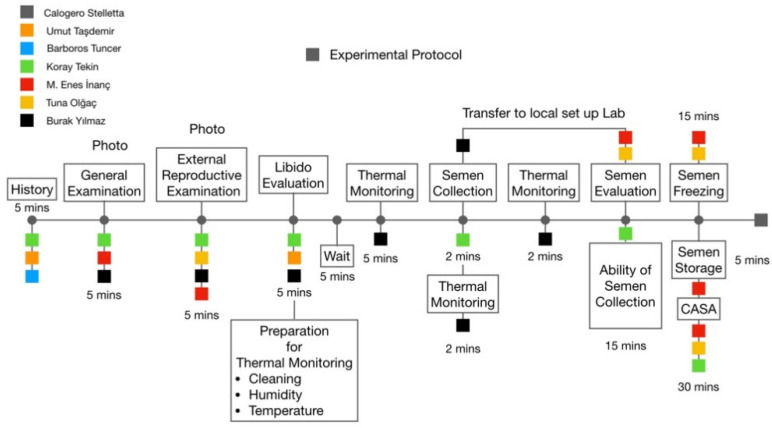
General experimental protocol across time.

The study was carried out at the beginning of spring season, under the environmental temperature was between 20–25 °C, the average percentage of the humidity was 59.0% during the experimental period. The experiment was performed in the Aksaray province of Turkey (381,962 N; 3359 E). Forty-six Aksaray Malakli dogs, aged between 2 to 7 years old, sexually active males were used and the dogs were grouped as ≤4 years and >4 years of age. Animals were not submitted to uniform nutritional conditions due to a different ownership. All the experimental protocol followed according to Figure 1 across time.

### 2.3. External Reproductive Examination

Body weights were measured using an electronic scale. Penis, prepuce, spermatic cord and scrotum were examined for the evidence of inflammation, abnormality, and thermoregulation of the testicle. Before semen collection, scrotum, testicle and epididymis were examined through palpation. The length, width, and height of both testicles were measured using a caliper, total testicular volume (cm^3^) was calculated using the following formula: length × width × height × 0.5236 [41] and grouped as ≤600 cm^3^ and >600 cm^3^.

### 2.4. Semen Collection

Semen samples were collected with manual manipulation by one experienced person without the presence of a bitch in estrus. Libido (1–5) and the ability of semen collection (0–2) were scored according to the response of the individuals during manipulation (Table 1). Total ejaculation time was calculated by summing up the duration of 1st, 2nd and 3rd fractions and expressed as seconds. Only the second, sperm-rich fraction of ejaculate was used for the subsequent semen processing. Fresh semen samples were immediately evaluated in a place where all the necessary equipment was set in a closed room.

### 2.5. Semen Assessment and Freezing

Semen collected from 42 individuals was evaluated and recorded macroscopically for volume, color, and pH. Immediately after collection, motility and concentration were determined using a phase-contrast microscope and a hemocytometer, respectively. The morphology assessment was done according to Inanc et al. [42], with “Sperm Blue©, Microptics, Barcelona, Spain” and the viability with eosin-nigrosine staining. The evaluation was performed by CASA morphology and viability with a manual counting program by SCA (SCA, Microptics, Barcelona, Spain). Ejaculates containing a minimum concentration of 150 × 10^6^ spermatozoa/mL and 70% progressive motility were used for cryopreservation. Tris-based extender (30.0 g of Tris, 17.0 g of citric acid, 12.5 g of fructose, 5% *v*/*v* glycerol, 20% *v*/*v* egg yolk with 1000 mL of distilled water at a pH of 6.8) was used for dilution (all chemicals were obtained by Sigma Aldrich, St. Louis, MI, U.S.). The ejaculates were diluted to a final concentration of 50 × 10^6^ spermatozoa/mL and were loaded into 0.25 mL volume straws. After 1.5 h of equilibration, the straws were placed horizontally 11 cm above the surface of nitrogen vapor, at −120 °C, for 15 min and were then plunged into the liquid nitrogen and stored at −196 °C. The straws were thawed in a water bath of 37 °C for 30 s. The post-thawed motility assessment was done with a computer aid sperm analyzer (SCA, Microptics, Barcelona, Spain) and the difference between the fresh and post-thawed motility (Δ motility) was calculated to determine the freezability of the samples.

### 2.6. Thermographic Monitoring

**Figure 2:**
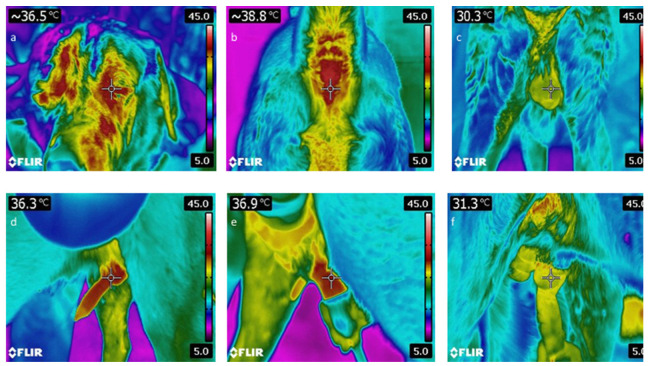
Thermal monitoring of body temperature and external reproductive organs before, during and after ejaculation. (**a**) Eye temperature before ejaculation; (**b**) Perianal temperature before ejaculation; (**c**) Scrotum before ejaculation; (**d**) Bulbus before ejaculation; (**e**) Penis during ejaculation; (**f**) Bulbus after ejaculation.

The surface temperatures of external genital organs from different regions of the penis and scrotum, as well as the eye and perianal area, were determined using a thermal camera (Flir Systems, E60, Limbiate, Italy) before, during and after ejaculation (Figure 2). Environmental temperature and humidity were collected by using a thermometer and hygrometer and were input into the system for each semen collection. Dogs were kept in an indoor area to avoid direct sun exposure during thermal monitoring. Before thermal scanning of the scrotum, the tail was held aside, and the scrotum was gently wiped with dry paper towels to remove any fecal matter and held on for 5 min. The surface temperatures of the regions of interest (ROI) were evaluated using the ThermaCam Researcher Basic software.

### 2.7. Statistical Analyses

Descriptive statistics for each variable were calculated and presented as “Mean ± SEM”. To evaluate the effects of age, BW, TTV and TET on the thermographic measures (eye average temperature, perianal, scrotum before ejaculation, delta eye, penis during ejaculation, scrotum after ejaculation) and Delta motility; a multivariate analysis of variance (MANOVA) model was applied. In the model, the main effects of age, BW, TTV and TET were included as independent variables, whereas the thermographic measures and delta motility were included as dependent variables. Due to the limited number of observations, any possible interaction terms were omitted from the model. Pillai’s trace test statistic was used for reporting the multivariate outcome. The Doornik–Hansen test was used to check multivariate normality and Box’s M test was used to check the homogeneity assumptions. After any significant effect, multivariate linear contrasts of each variable were tested as a follow-up procedure to find the nature of the differences. A probability value of less than 0.05 was considered significant unless otherwise noted. Pearson’s and Spearman correlation analyses were used to evaluate the relationship between thermographic measurements and semen characteristics in consideration with the distribution properties of the variables. A probability value of less than 0.05 was considered significant unless otherwise noted. All statistical analyses were performed by using Stata 12.1 MP4 statistical software.

## 3. Results

The semen was collected with a 91.2% success rate. However, some individuals could not meet the further criteria for the sperm cryopreservation procedure due to their increased dead or abnormal spermatozoa rate. The mean behavioral, spermatological and thermographic measurements were presented in Table 2. According to experimental experiences, younger males had better body condition, more consistent and elevated scrotal sac, thus had lower TTV, along with firm and consistent testicular parenchyma. The TET, fraction volumes and motility were higher in older males. However, there were not any differences determined in post-thaw motility between the age groups.

The overall multivariate test has shown the effect of classes of age (*p* < 0.001), BW (*p* < 0.001), TTV (*p* < 0.001), TET (*p* < 0.001) and CON (*p* < 0.001) on thermographic measurements of scrotum, penis, eye and perianal area as well as the Δ motility (Table 3).

To find the nature of the differences, multivariate linear contrasts of each variable were tested as a follow-up procedure (Table 4). According to that, better freezability values were obtained from heavier males (*p* < 0.05), dogs that have more consistent testicles (*p* < 0.01) as well as quicker ejaculate responders (*p* < 0.001). However, there was not any effect of age alone on the freezability of sperm. The initial scrotal temperature before the ejaculation was lower in older (*p* < 0.05) and heavier dogs (*p* < 0.001), with lower TTV (*p* < 0.001) and quicker ejaculate responders (*p* < 0.01). Penis temperature during the ejaculation showed similar patterns to the initial scrotal temperature regarding all classes, except for the age (*p* > 0.05) and CON classes (*p* < 0.001). More interestingly, the sperm count was found higher in dogs that had a lower eye temperature prior to the ejaculation (*p* < 0.01); similarly, lower scrotal temperature following the ejaculation (*p* < 0.01) and conversely the higher penile temperature during the ejaculation (*p* < 0.001). In males with a sperm count lower than 300 million per ejaculate, the temperature of the eye was hotter prior to the ejaculation, the penis was colder during the ejaculation and the scrotum was hotter following the ejaculation compared to those with a higher sperm count (Table 4). Apart from that, the temperature of the eye prior to the ejaculation was lower in older and lighter dogs, with less consistent testicles and slower ejaculate responders (*p* < 0.001).

Fresh motility was higher in ejaculates collected from increased penis temperature during the ejaculation (r = 0.42, *p <* 0.05). Perianal temperature has shown a positive correlation with semen volume (r = 0.31, *p <* 0.05). Apart from that, as the temperature of the bulbus and penis increased prior to ejaculation, the ability to collect semen was decreased. Similarly, sperm abnormalities were lower in the ejaculate of dogs that had a higher temperature of scrotum, bulbus and penis (Table 5).

## 4. Discussion

In the present study, thermal monitoring of the male dogs through the ejaculation process was performed to define the thermal patterns of the reproductive organs and their link with semen quality and freezability. Thermal patterns of the eye and perianal region were recorded as stress indicators to reveal the behavioral response of the animals to the manipulation. Since there are several factors affecting this thermal response, the collected data were evaluated regarding various physiological and reproductive traits of the subjected animals such as age, BW, TTV, TET and CON.

It is known that testicular functionality in mammalian species with extra-corporal testicles depends on the intra-testicular temperature, which is approximately 2.5–5.3 °C lower than the body temperature (bull: [43,44,45,46]). Henning et al. [47], reported a mean of 32.8 °C ± 0.4 °C scrotal surface temperature in seven mature dogs. In the present study, the mean scrotal temperature measured with IRT was 31.13 °C and increased 1.49 °C following the sperm collection, still remaining lower than the body temperature. According to our results, both initial scrotal temperature before the ejaculation and the Δ motility varied regarding the body weight, total testicular volume and total ejaculation time of the dogs. Contrasting with the findings of Ramires-Neto et al. [21], where the young stallions had a lower scrotal surface temperature than the older ones, the scrotal temperature before ejaculation was higher in younger males, although the sperm freezability was not affected by the age of the animals since the semen was collected from a dog population with a narrow range of age (2–7 years of age). Apart from that, better sperm freezability was obtained from heavier males, lower testicular volume and shorter length of ejaculation. The duration of fractions in dogs were 5–90 s, 5–300 s and 60 s to 20 min respectively with an average of 481 s of total ejaculation time [37]. In the present study, the total ejaculation time ranged between 703 to 1095 s with an average of 823 s, which was in the physiological range but higher than the average duration. It is known that oxidative stress can cause swelling and cell membrane fluidity, interfere with ionic gradients, which could lead to an inflammation of the tissue resulting in swollen testicles. It should also be kept in mind that the ultrasonographic examination of the testicle was not conducted on these animals, that is why the increase of the testicular volume and the related impairment in sperm freezability might have resulted from underlying causes such as testicular masses, which might not be palpable. Actually, these findings introduce another discussion about the relations of scrotal thermoregulation with the aging and physical condition of the animals. Based on our results, the physical well-being reflected as BW and TTV had a significant relationship with semen freezability while age did not. Therefore, it would be better to accept the fact that the males who kept their physical well-being regarding reproductive function had no difference in sperm freezability when only aging was taken into account.

There are only a few studies focused on the relationship between testicular temperature and sperm quality in animals. It is reported that long-term scrotal insulation could affect sperm quality due to disruption in spermatogenesis [44]. The common findings in ejaculates related to the increase at scrotal surface temperature are a reduced sperm output (ram: [48,49]; lama: [50]), a lower proportion of motile sperm (bull: [45,51]; dog: [47] ram: [48,49]; lama: [50]), and a more frequent occurrence of spermatozoa with altered morphology (bull: [16,18,51,52], ram: [48]; lama: [50]). Contrary to what was expected, the results of the current study demonstrated that there was a moderate negative correlation between the increase in the scrotal temperature and the sperm abnormalities in the concurrent ejaculate. The impairment in semen quality due to an increase in the scrotal temperature is observed after long-term exposure and particularly following insulation of the testicle. The normal morphology of the sperm is preserved for a few days even though the testicular temperature increases since the sperm in the epididymis is not majorly affected [53]. It is stated that a consistent interval is present from the elevation of testicular temperature to the detection of sperm defects in the ejaculate [19]. Henning et al. [47] stated that short-term scrotal hyperthermia did not affect sperm quantity and quality in dogs, contrary to other species. Besides, in the current study, the scrotal temperatures varied between 31.0–34.8 °C, which is considered in the physiological range. During the ejaculation, the contractions of the testicular capsule could lead to a diminishing of the vascularity in the testicular area that could explain the decrease in the temperature following ejaculation [26]. Although no relationship was observed between sperm concentration and scrotal temperature prior to ejaculation, however, the concentration was significantly higher in dogs that had a higher scrotal temperature following ejaculation. As the scrotal temperature response seems to have a relation to sperm quality, it is necessary to understand the underlying effects. In contrast to the reports on other species, the semen quality of the dog is less affected by the scrotal temperature variations and looks like they can compensate through scrotal thermoregulation.

The sperm count was higher with a lower scrotal temperature following ejaculation and a higher fresh motility was obtained with higher penis temperature during the ejaculation. Scrotal hyperthermia is a stressor for testicular and epididymal cells [47]. A non-physiological rise in the temperature induces changes in gene expression, indicating signs of hypoxia and oxidative stress in mouse testicular tissue [54]. When the higher demand for oxygen supply in the reproductive tissue is not compensated, vasodilatation of the testicular artery in the pampiniform plexus is rendered an insufficient mechanism [55]. In canines, this instant reaction could be explained with a muscle reaction which could determine the quality and duration of the response as ejaculation, and thus semen quality. More interestingly, as the temperature of the bulbus prior to ejaculation and penis both prior and during ejaculation increased, the percentage of sperm defects in the concurrent ejaculation was decreased.

The eye and face for IRT monitoring activity, is reported by different authors, describing the relationship of these surface temperatures with hyperthermia other than the variation during behavioral experiences and stressful conditions [56,57]. According to Beaver [58], 60% of animals brought to veterinary practice are stressed, fearful and submissive. Evolutionarily, during ejaculation, dogs are more prone to opponent threats and they are under the effect of the sympathetic branch of the autonomic nervous system, which prepares the animals for the fight reaction [25]. In order to look out for the environmental stimulations, the copulation tie occurs in a position in which the animals turn their rear ends to each other. The relationship between stress and body temperature could be explained through the activation of the hypothalamic–pituitary–adrenal axis as the increase in catecholamines and cortisol levels and responses of blood flow, heat production is expected [28,59]. The results of the current study showed that the temperature of the eye was statistically lower in older and lighter males and in the dogs that had smaller testicular volume and slower ejaculate responders. Besides, the sperm count was higher in those with a lower eye temperatures prior to ejaculation. Divero et al. [60] studied rescue dogs at an ambient temperature of −10 °C, following their transportation via helicopter and lowering by a harness from the helicopter, and reported that an increase of 0.58 °C of body temperature has been detected and remained relatively constant through the 10 min of search activity. Similarly, it has demonstrated that search and rescue dogs peaked their rectal temperature and the temperature remained stable for the 30-min recovery period [61]. In the present study, although the perianal temperature was not significantly changed related to the physiological and reproductive traits of the animals, eye temperature differed significantly between different classes of these parameters. Stewart et al. [33] reported that, as the stress factors emerge, they initiate the acute phase with an instant drop of the eye temperature during the following seconds as a sympathetic response due to peripheral vasocontraction. In the chronic phase, as the stress factor remains, a cortisol release maintains from minutes to hours, resulting in thermogenic reactions in tissue metabolism, that increase the eye temperature [34,62]. It is reported that both eye and ear temperature are accurately relatable to rectal temperature following the hyperthermal changes due to exercise. However, during the resting period, the eye temperature related to rectal temperature was less accurate than the ear temperature. It should also be kept in mind that the stress factors may not always be related to behavioral changes due to variability in gender, breed, experience or type of stressful situation [63,64,65,66], and observation of spontaneous behavior is needed to interpret physiological data [67,68]. In that vein, Pasing et al. [68] have reported that stallions increased their heart rate in response to semen collection (*p* < 0.001); however, it was seen that stallions remained calmer during the collection. It can be hypothesized that, as the positive correlations and relationships between the increase of the body temperature and reproductive traits demonstrated, this elevation might not arise from a particular stress factor but may reflect anticipation or instant excitement.

## 5. Conclusions

The thermal monitoring can be utilized as an auxiliary tool to determine the behavioral status of the breeder candidate prior to semen retrieval. Especially, before the semen collection, the candidate should be adapted to the foreign environment, which may affect the sperm quality of the individual. Here, the thermal response of the procedure can be determined by performing eye and rectal measurements before semen collection for behavioral and the penis, bulbus and scrotal measurements during erection for ejaculation response. The compliance of reproductive behaviors with sperm retrieval could be converted into parametric values by thermal imaging. It can be used as an important tool for the evaluation of erectile function, especially in stallions, bulls and other species, where the examination of external reproductive organs is risky without sedation, but physiological erection cannot be achieved after sedation. Besides, the Malaklı dog breed could be a potential model for human male infertility considering anatomic and physiologic similarities. Finally, it can be applied as an auxiliary tool for the pre-diagnosis of some abnormal subclinical conditions with a temperature increase above normal values in the external reproductive organs. Therefore, clinical/practical use needs validation of this technique and physiological–pathological maps should be created with a thermal camera for further investigation.

## Figures and Tables

**Table 1:** The evaluation scale of reproductive behavioral parameters.

Libido Sexualis		Ability of Semen Collection	
Secretion of 1st fraction immediately	5	Non-responsive (<5 min)	0
Secretion of 1st fraction 0–45 s.	4	Responsive (>5 min)	1
Secretion of 1st fraction 45 s–1.5 min.	3	Hypersensitive (>1 min)	2
Secretion of 1st fraction 1.5–5 min.	2		
Secretion of 1st fraction >5 min.	1		

**Table 2:** The average value of behavioral, spermatological and thermographic parameters.

Parameters	*n*	Mean	Standard Deviation	Minimum	Maximum	Median
Libido Score (1–5)	46	4.77	0.57	2.00	5.00	5.00
Ability of Semen Collection (0–2)	46	1.60	0.73	0.00	2.00	2.00
Semen Volume (mL)	42	11.74	6.04	4.60	26.50	9.80
1st Fraction Volume (mL)	42	2.84	1.86	0.50	10.00	2.30
2nd Fraction Volume (mL)	42	2.45	1.25	0.80	6.00	2.00
3rd Fraction Volume (mL)	42	6.45	4.88	0.50	20.00	5.00
1st Fraction pH	42	5.97	0.24	5.50	6.50	5.90
2nd Fraction pH	42	5.80	0.22	5.30	6.30	5.80
3rd Fraction pH	42	5.84	0.23	5.40	6.80	5.80
Total Ejaculation Time (s)	42	823.72	109.24	703.00	1095.00	775.00
Dead Spermatozoa (%)	42	17.70	7.88	7.19	34.44	15.35
Acrosome Abnormality (%)	42	0.49	0.75	0.00	2.77	0.00
Head Abnormality (%)	42	2.39	4.84	0.00	31.27	1.60
Midpiece Abnormality (%)	42	2.35	3.64	0.00	17.94	1.00
Tail Abnormality (%)	42	7.67	7.92	0.00	32.98	5.04
Cytoplasmic Droplet (%)	42	2.70	4.38	0.00	16.60	1.03
Total Abnormal Sperm (%)	42	15.62	11.88	1.04	45.09	11.65
Fresh Motility (%)	42	86.05	4.39	75.00	90.00	88.00
Post-thaw Motility (%)	42	33.86	14.78	11.20	67.51	33.11
Delta Motility (%)	42	−52.34	15.63	−12.49	−78.03	−53.65
Eye Before EJA (°C)	42	34.42	2.86	27.30	40.20	34.40
Perianal Before EJA (°C)	42	35.30	2.55	28.10	38.90	35.90
Scrotum Before EJA (°C)	42	31.13	4.38	29.11	34.80	31.05
Scrotum After EJA (°C)	42	32.62	5.58	28.30	35.50	31.25
Bulbus Before EJA (°C)	42	33.60	2.68	25.90	36.70	34.20
Penis Before EJA (°C)	42	31.91	3.45	24.60	36.10	33.10
Penis During EJA (°C)	42	29.16	3.72	18.40	34.70	29.81

**Table 3:** The results of the overall multivariate test (*n* = 46).

	Statistics *	df	F (df1, df2)		*p*
Model	3.205	5	35.44 (35, 170)		<0.001
Age	0.987	1	345.12 (7, 30)		<0.001
Body Weight	0.958	1	98.03 (7, 30)		<0.001
Total Testicular Volume	0.923	1	51.09 (7, 30)		<0.001
Total Ejaculation Time	0.905	1	40.83 (7, 30)		<0.001
Concentration	0.954	1	90.53 (7, 30)		<0.001
Residual		36			
Total		41			

* Pillai’s trace statistic.

**Table 4:** The effect of independent values (Age, BW, TTV, TET and CON) on each selected dependent variable.

Dependent Variables	Independent Variables	Category	Margin	Std. Error	dy/dx *	Std. Error	*p*
∆ Motility	Age	≤4	−44.64	2.65	−7.36	5.48	0.325
		>4	−52.00	6.66			
	BW	≤75	−54.27	4.39	17.66	7.81	0.024
		>75	−36.61	3.71			
	TTV	≤600	−35.98	4.65	−18.05	7.28	0.014
		>600	−54.03	4.06			
	TET	≤800	−29.83	6.02	−21.05	7.28	0.004
		>800	−50.88	2.92			
	CON	≤300	−47.35	5.37	2.07	5.76	0.759
		>300	−45.27	3.04			
Scrotum Before EJA	Age	≤4	29.18	0.36	−2.2	1.01	0.029
		>4	26.98	0.89			
	BW	≤75	33.26	0.59	−9.34	1.05	0.001
		>75	23.92	0.63			
	TTV	≤600	26.60	0.62	4.03	0.98	0.001
		>600	30.64	0.55			
	TET	≤800	26.57	0.81	2.93	0.98	0.003
		>800	29.51	0.39			
	CON	≤300	29.71	0.72	−1.26	0.91	0.166
		>300	28.45	0.41			
Penis During EJA	Age	≤4	30.17	0.20	−0.25	0.57	0.671
		>4	29.92	0.51			
	BW	≤75	34.76	0.34	−9.70	0.60	0.001
		>75	25.04	0.36			
	TTV	≤600	26.45	0.36	6.72	0.56	0.001
		>600	33.18	0.31			
	TET	≤800	26.47	0.46	4.81	0.56	0.001
		>800	31.27	0.22			
	CON	≤300	27.87	0.41	3.16	0.52	0.001
		>300	31.03	0.23			
Eye Before EJA	Age	≤4	36.38	0.13	−2.47	0.35	0.001
		>4	33.91	0.32			
	BW	≤75	34.42	0.21	3.25	0.37	0.001
		>75	37.67	0.22			
	TTV	≤600	37.66	0.22	−3.09	0.35	0.001
		>600	34.57	0.19			
	TET	≤800	38.21	0.29	−2.93	0.35	0.001
		>800	35.27	0.13			
	CON	≤300	36.70	0.26	−1.02	0.32	0.002
		>300	35.68	0.14			
Perianal Before EJA	Age	≤4	36.79	0.24	0.03	0.69	0.967
		>4	36.82	0.62			
	BW	≤75	36.72	0.41	0.17	0.72	0.811
		>75	36.89	0.43			
	TTV	≤600	36.83	0.43	−0.04	0.68	0.954
		>600	36.79	0.37			
	TET	≤800	36.08	0.56	0.95	0.67	0.158
		>800	37.03	0.27			
	CON	≤300	37.44	0.49	−0.89	0.63	0.154
		>300	36.55	0.28			
Scrotum After EJA	Age	≤4	29.69	0.47	−2.27	1.33	0.088
		>4	27.42	1.18			
	BW	≤75	30.09	0.78	−1.64	1.39	0.238
		>75	28.45	0.84			
	TTV	≤600	30.28	0.83	−1.75	1.31	0.180
		>600	28.52	0.72			
	TET	≤800	30.25	1.07	−1.24	1.29	0.340
		>800	29.02	0.52			
	CON	≤300	32.01	0.96	−3.77	1.29	0.002
		>300	29.69	0.47			

* dy/dx for factor levels is the discrete change from the base level; BW = body weight; TTV = total testicular volume; TET = total ejaculation time; CON = sperm concentration; EJA = ejaculation.

**Table 5:** The correlation indices among thermal parameters and semen characteristics.

(*n* = 42)	Eye before EJA	Perianal before EJA	Scrotum before EJA	Scrotum after EJA	Bulbus Before EJA	Penis before EJA	Penis during EJA
Libido scoring	0.336 *						
The ability of semen collection					−0.476 *	−0.508 **	
Semen volume		0.306 *					
Dead Spermatozoa							−0.386 *
Acrosome abnormality					−0.440 *		
Head abnormality				−0.391 *	−0.576 **		−0.349 *
Total Head Abnormality					−0.714 **	−0.422 *	−0.476 **
Midpiece abnormality			−0.361 *				−0.537 **
Total Sperm Defects					−0.516 *		
Fresh Motility							0.416 *

* Correlation is significant at the 0.05 level (2-tailed). ** Correlation is significant at the 0.01 level (2-tailed). EJA = ejaculation.

## Data Availability

The data presented in this study are available on request from the corresponding author. The data are not publicly available due to the agreement with funding bodies.
